# Alterations of Fatty Acid Profile May Contribute to Dyslipidemia in Chronic Kidney Disease by Influencing Hepatocyte Metabolism

**DOI:** 10.3390/ijms20102470

**Published:** 2019-05-18

**Authors:** Aleksandra Czumaj, Tomasz Śledziński, Juan-Jesus Carrero, Piotr Stepnowski, Malgorzata Sikorska-Wisniewska, Michal Chmielewski, Adriana Mika

**Affiliations:** 1 Department of Pharmaceutical Biochemistry, Faculty of Pharmacy, Medical University of Gdansk, 80-211 Gdansk, Poland; aczumaj@gumed.edu.pl (A.C.); adriana.mika@ug.edu.pl (A.M.); 2 Medical Epidemiology and Biostatistics (MEB), Karolinska Institutet, SE-171 77 Stockholm, Sweden; Juan.Jesus.Carrero@ki.se; 3 Department of Environmental Analysis, Faculty of Chemistry, University of Gdansk, 80-308 Gdansk, Poland; piotr.stepnowski@ug.edu.pl; 4 Department of Nephrology, Transplantology and Internal Medicine, Faculty of Medicine, Medical University of Gdansk, 80-214 Gdansk, Poland; gosia.sikorska@gmail.com (M.S.-W.); chmiel@gumed.edu.pl (M.C.)

**Keywords:** chronic kidney disease, fatty acids, lipids, lipogenesis, hypertriglyceridemia, hepatocyte

## Abstract

Chronic kidney disease (CKD) is associated with atherogenic dyslipidemia. Our aim was firstly to investigate patterns of fatty acids (FA) composition through various stages of CKD, and secondly, to evaluate the effect of CKD-specific FA disturbances on the expression of genes related to lipid metabolism at a cellular level. Serum FA composition was analyzed in 191 patients with consecutive severity stages of CKD, and 30 healthy controls free from CKD. Next, HepG2 human hepatic cells were treated with major representatives of various FA groups, as well as with FA extracted from a mix of serums of controls and of CKD stage 5 patients. Across worsening stages of CKD severity, there was an increasing monounsaturated FA (MUFA) content. It was associated with a concomitant decrease in n-3 and n-6 polyunsaturated FA. The incubation of hepatocytes with FA from CKD patients (compared to that of healthy subjects), resulted in significantly higher mRNA levels of genes involved in FA synthesis (fatty acid synthase (*FASN*) increased 13.7 ± 3.5 times, stearoyl-CoA desaturase 1 (*SCD1*) increased 4.26 ± 0.36 times), and very low density lipoprotein (VLDL) formation (apolipoprotein B *(ApoB*) increased 7.35 ± 1.5 times, microsomal triacylglycerol transfer protein (*MTTP*) increased 2.74 ± 0.43 times). In conclusion, there were progressive alterations in serum FA composition of patients with CKD. These alterations may partly contribute to CKD hypertriglyceridemia by influencing hepatocyte expression of genes of lipid synthesis and release.

## 1. Introduction

Chronic kidney disease (CKD) is accompanied by lipid disorders due to metabolic alterations associated with the retention of uremic solutes, as well as to dietary restrictions and impaired food intake resulting from chronic intoxication [1]. Dialysis does not normalize the lipid profile, and in patients after renal transplantation it may even further aggravate, mainly because of the immunosuppressive treatment. CKD-dyslipidemia likely contributes to increased cardiovascular risk [1]. 

Lipids count over 1.6 million species and most of them contain fatty acids (FA) in their structure [2]. Serum FA concentration and FA composition has a significant effect in maintaining the metabolic balance in the organism. Disturbed blood FA profile is associated with oxidative stress, lipotoxicity and hypertriglyceridemia. Patients suffering from CKD are potentially prone to FA disorders due to metabolic alterations associated with the retention of uremic solutes, as well as to dietary restrictions and impaired food intake resulting from chronic intoxication.

Various studies, including ours, have reported alterations in the proportion of particular serum FA groups across more severe CKD stages, particularly a progressively lower n-3 polyunsaturated fatty acids (PUFA) content and higher monounsaturated fatty acids (MUFA) content [3,4,5,6]. The consequences of these changes are not known. Studies outside nephrology suggest that FA can modulate a number of cellular pathways involved in lipid and lipoprotein metabolism. In fact, they may regulate gene expression, and signal transduction cascades, e.g., through transcription factors including sterol regulatory binding proteins (SREBPs), peroxisome proliferator-activated receptors (PPARs), hepatocyte nuclear factor 4α (HNF4α), liver-x receptors (LXR) and nuclear factor κB (NFκB) [7,8,9,10,11]. They also play important roles in numerous physiological processes, including energy metabolism (secretion of insulin, glucagon and incretins) and inflammatory responses (cytokine production) [12]. 

While it is well-established that dietary FA can impact on the regulation of genes involved in lipid metabolism [13,14], it is conceivable that CKD-induced changes in serum FA composition may also play a role at this level. The aim of this study was firstly to investigate patterns of FA composition through various stages of CKD. Secondly, given that liver is the major organ responsible for lipid metabolism, we studied the effect that these CKD-specific FA disturbances might have on the expression of genes related to lipid metabolism in hepatocytes. 

## 2. Results

### 2.1. Clinical Data

Characteristics of the included patients with CKD, undergoing hemodialysis (HD) or peritoneal dialysis (PD), and after renal transplantation (Tx) are presented in Table 1. Across worsening CKD stages, we noted increased concentrations of triacylglycerols (TAG), glucose, insulin, homeostatic model of insulin resistance (HOMA IR), C-reactive protein (CRP) and K^+^. In addition, hemoglobin, albumin and HDL cholesterol were decreased, while total and LDL cholesterol remained fairly stable (Table 1).

**Figure 1:**
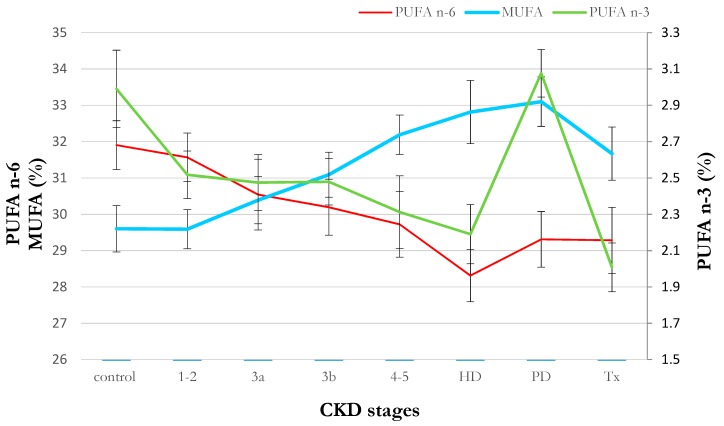
Serum proportion of n-3, n-6 polyunsaturated fatty acids (PUFA) and monounsaturated fatty acids (MUFA) in chronic kidney disease (CKD) patients with consecutive stages of disease. Data are shown as mean ± SEM. * *p* < 0.01; ^#^ *p* < 0.05 comparing to healthy control group. CKD—chronic kidney disease; HD—hemodialysis; PD—peritoneal dialysis; Tx—renal transplantation; PUFA—polyunsaturated fatty acids; MUFA—monounsaturated fatty acids.

The serum FA profile showed significant alterations in the composition of many FA families across worsening stages of CKD (Table 2). The most prominent alterations were an increase in MUFA content and a decrease in n-3 and n-6 PUFA (Figure 1). Following a multivariable general linear model (GLM) analysis (adjusted by age, sex and diabetes mellitus), CKD stage emerged as a significant predictor of MUFA and PUFA proportions. Dialysis therapies did not seem to modify this pattern, except for the n-3 PUFA level in PD subjects (Figure 1). Obtained results also did not show considerable associations between the consumption of food products rich in MUFA, n-3 PUFA or n-6 PUFA and serum content of these FA in CKD patients (Table S1). Lipid lowering medications have not altered patients FA profile significantly.

### 2.2. Cell Studies In Vitro

To examine if the observed alterations in the serum FA composition in CKD patients had an impact on dyslipidemia related genes, we treated human HepG2 hepatocytes with the main MUFA, n-3 and n-6 PUFA representatives—oleic acid (OA), docosahexaenoic acid (DHA) and arachidonic acid (AA), respectively. 

**Figure 2:**
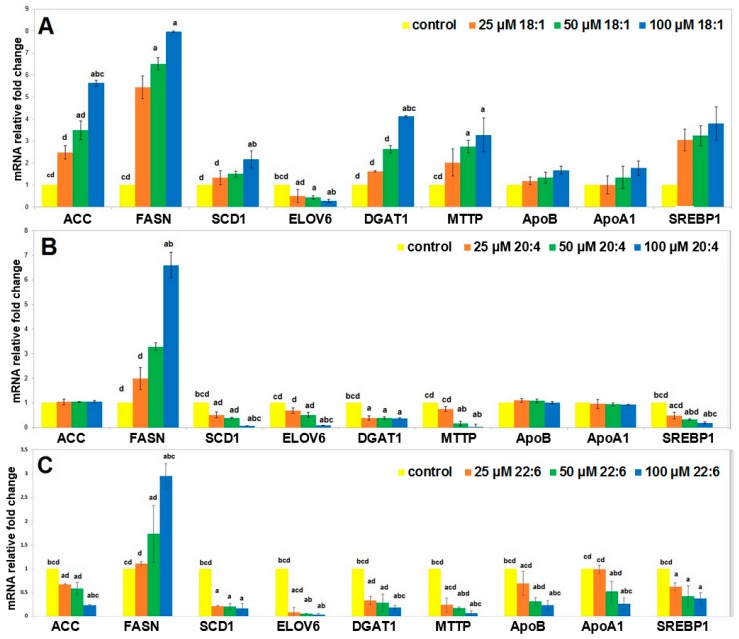
mRNA levels for selected genes expressed in HepG2 cells supplemented with various concentrations of oleic acid (OA; **A**), arachidonic acid (AA; **B**) and docosahexaenoic acid (DHA; **C**). Data are shown as mean ± SEM. Groups significantly different by one-way ANOVA (*p* < 0.05): a—*p* < 0.05 compared to the control; b—*p* < 0.05 compared to the cells treated by 25 µM FA; c—*p* < 0.05 compared to the cells treated by 50 µM FA and d—*p* < 0.05 compared to the cells treated by 100 µM FA. Data are presented as means ± SEM. *ACC*—acetyl-coenzyme A carboxylase, *FASN*—fatty acid synthase, *SCD1*—stearoyl-CoA desaturase, *ELOVL6*—fatty acid elongase 6, *DGAT1*—diacylglycerol O-acyltransferase 1, *MTTP*—microsomal triglyceride transfer protein, *ApoB*—apolipoprotein B, *ApoA1*—apolipoprotein A1, *SREBP1*—sterol regulatory element-binding protein 1.—control; –25 µM FA; –50 µM FA; –100 µM FA.

Incubation with OA resulted in a significant increase in mRNA levels of genes encoding enzymes of fatty acid synthesis and desaturation (acetyl-CoA carboxylase (*ACC*), fatty acid synthase (*FASN*) and stearoyl-CoA desaturase (*SCD1*)), TAG synthesis (diacylglycerol O-transferase (*DGAT1*)), as well as microsomal TAG transfer protein (*MTTP*) involved in very low density lipoprotein (VLDL) formation (Figure 2A). There was also a trend towards an increased mRNA level of *SREBP1*—transcription factor promoting lipid synthesis, and apolipoproteins A1 (*ApoA1*) and B (*ApoB*). In contrast, mRNA levels of FA elongase 6 (*ELOVL6*) decreased (Figure 2A). All these changes appeared to be dose dependent. 

Incubation with AA led to a decrease in *SCD1, ELOVL6, DGAT, MTTP* and *SREBP1*, it did not change the expression of *ACC*, and apolipoproteins, and increased *FASN* mRNA levels (Figure 2B). Incubation of hepatocytes with DHA resulted in generally the same changes of genes expression as the treatment with AA (Figure 2C).

Summarizing this first experiment, OA generally increased the expression of genes related to liver lipid synthesis and release (except *ELOVL6*), whereas AA and DHA decreased or did not influence the expression of these genes (except *FASN*).

### 2.3. Cell Studies Ex Vivo

As a next step, we extracted FA from pooled serum of 12 CKD stage 5 patients and of 12 healthy controls, and we incubated hepatocytes with such FA preparation. The composition of the CKD- and control-FA preparations was evaluated by GC-MS and presented in Table 3. FA preparations extracted from CKD patients had higher MUFA and lower n-3 as well as n-6 PUFA content in comparison to those of control individuals.

**Figure 3:**
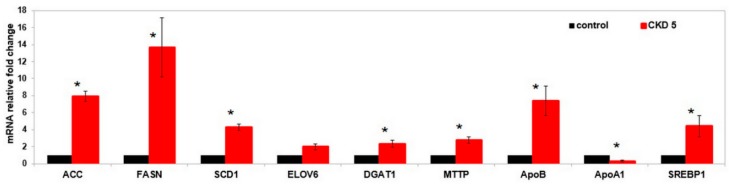
mRNA levels for selected genes expressed in HepG2 cells supplemented with fatty acids (FA) from control patients (control) and CKD stage 5 patients (CKD 5) serum. * *p* < 0.05 compared to the control. Data are presented as means ± SEM. *ACC*—acetyl-coenzyme A carboxylase, *FASN*—fatty acid synthase, *SCD1*—stearoyl-CoA desaturase, *ELOVL6*—fatty acid elongase 6, *DGAT1*—diacylglycerol O-acyltransferase 1, *MTTP*—microsomal triglyceride transfer protein, *ApoB*—apolipoprotein B, *ApoA1*—apolipoprotein A1, *SREBP1*—sterol regulatory element-binding protein 1.

**Figure 4:**
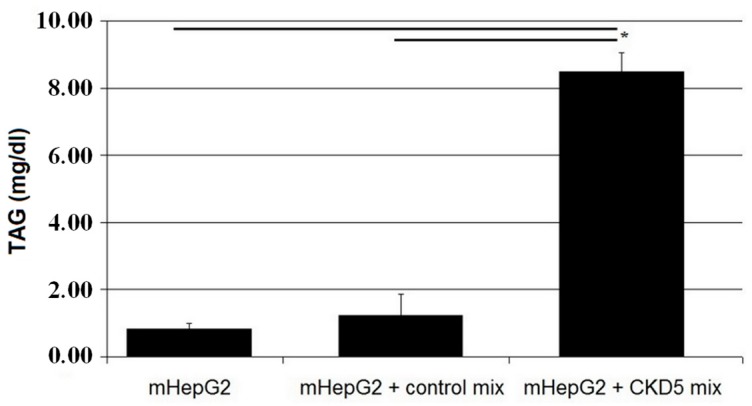
Concentration of TAG in culture medium after 48 h incubation with FA mixtures. Data are shown as mean ± SEM, * *p* < 0.05.

Compared to the FA mixture of healthy subjects, incubation of HepG2 with FA from CKD patients resulted in significantly increased mRNA levels of genes involved in fatty acid synthesis, desaturation, elongation and VLDL formation, as well as the *SREBP1* gene (Figure 3). On the other hand, *ApoA1* mRNA level was lower after incubating with CKD FA mixture. Finally, the concentration of TAG in the culture medium after 48 h culture of cells treated with the CKD FA mixture was approximately six-fold higher than following incubation with FA mixture from controls (Figure 4).

## 3. Discussion

Although previous studies have evaluated disorders in FA profile in patients with CKD, they have been usually limited to dialysis patients only [3,4,5,15]. In contrast, our study provides a comprehensive overview of changes in serum FA composition across the natural course of CKD progression. We show that across worsening stages of CKD severity, there was an increasing MUFA and gradually decreasing n-3 and n-6 PUFA content. Our recent study revealed that increased serum MUFA in CKD patients is associated with various risk factors of cardiovascular disease, primarily with hypertriglyceridemia [6]. Kidney transplantation did not result in full normalization of these derangements. 

The novelty of our study is the evaluation of the impact of these CKD-specific FA alteration on lipid metabolism. We used an in vitro model of hepatocytes, given that the liver is the main organ involved in lipid metabolism, observing that oleic acid, the major MUFA representative, resulted in an increased expression of lipogenic genes (*ACC, FASN, SCD1, DGAT1*), as well as genes related to VLDL production and release (*ApoB, MTTP*). Such changes have been reported to result from an increased expression of *SREBP1*—an upstream regulator of lipogenic genes [16]. It should be noted that increased expression of *SREBP1* has been found in adipose tissue of rats with experimental renal failure in our previous studies [17]. Our results are consistent with these obtained recently by Patel et al. [18] who also found increased mRNA levels of *SPREBP1*, *ACC* and *FASN* in hepatocytes treated with OA, although these authors used much higher OA concentrations in culture (1 mM), in comparison to our study (25–100 µM). 

In contrast, hepatocyte treatment with arachidonic acid (major n-6 PUFA representative) as well as docosahexaenoic acid (major n-3 PUFA representative), decreased or did not materially change mRNA levels of most of these genes. Only *FASN* mRNA level increased significantly after treatment with 100 µM AA, a finding that seemed counterintuitive, given the behavior of the rest of the genes in the same pathway. Decreased hepatic de novo lipogenesis following n-6 PUFA treatment was also described by Caputo et al. [19] and Mater et al. [20]. 

**Figure 5:**
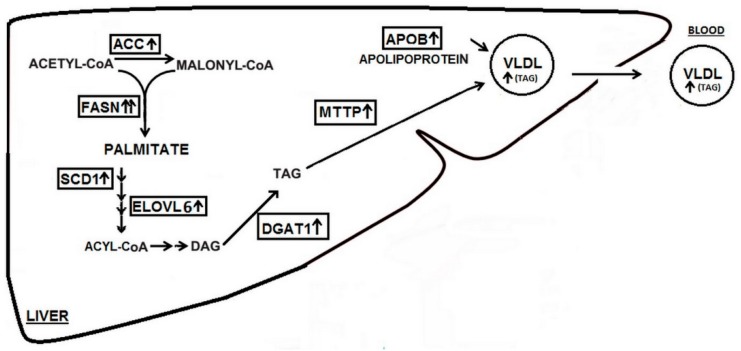
Scheme illustrating the plausible effects of fatty acids extracted from chronic kidney disease (CKD) patients’ serum on the expression of genes associated with lipid synthesis and release in hepatocyte. *ACC*—acetyl-coenzyme A carboxylase, *FASN*fatty acid synthase, *SCD1*—stearoyl-CoA desaturase, *ELOVL6*—fatty acid elongase 6, *DGAT1*—diacylglycerol *O*-acyltransferase 1, *MTTP*—microsomal triglyceride transfer protein, *ApoB*—apolipoprotein B, DAG—diacylglycerol, TAG—triacylglycerol, VLDL—very low density lipoprotein.

Altogether, this evidence suggests that CKD-specific FA alterations might (by influencing liver metabolism) increase lipid production and release, leading to CKD-associated hypertriglyceridemia. However, an important limitation in performing this kind of experiments is the use of a single FA for cell incubation rather than the whole FA spectrum that contains more than 30 different FA [21]. Since each FA can influence liver cells, the CKD-specific alterations induced by the FA profile should ideally be considered globally. To tackle this, we used a pool preparation of FA isolated from serum of healthy subjects and patients with CKD stage 5. This experiment revealed that the pathological serum FA profile of CKD patients, including increased MUFA and decreased PUFA, might induce lipid synthesis and release in liver. Finally, increased triacylglycerol concentrations in culture media from cells treated with FA from CKD patients confirmed the functional end-point of changes in lipogenic genes expression. A schematic representation of our observations and working hypothesis is shown in Figure 5.

Considering that MUFA are the main FA in triacylglycerols included in VLDL [22,23], we speculate that a self-perpetuating mechanism arises, in which the more MUFA in the patient’s serum, the greater the production of triacylglycerols in the liver. This effect might contribute to hypertriglyceridemia, constantly observed in CKD patients. Increased triglyceride concentration is an acknowledged risk factor for cardio-vascular disease in the general population. Therefore, a gradual increase in MUFA across the CKD stages, combined with low PUFA content, demonstrated in the present study, might result in an augmented risk of cardio-vascular complications. Dietary modifications could diminish this risk, although this remains to be determined.

Treatment of hepatocytes with FA from CKD patients resulted also in an increased *SREBP1* mRNA level, confirming its role in the induction of liver lipogenesis in CKD. Other authors reported that SREBP1 activity in the liver can be modulated by various FA [24]. The role of SREBPs in CKD dyslipidemia was also reported earlier by our group, on the basis of an experimental renal failure in animal models [16]. The upregulation of lipogenic enzymes was also reported in a rat renal failure model by Jin et. al. [25]. 

The study has limitations that ought to be underlined. First, we include a cross-sectional evaluation, therefore results present FA disorders across various CKD stages with no inference on temporality within disease progression or causation. A study design in which the same patients are followed and repeatedly sampled throughout their disease progression would have been preferred, but taken potentially decades to complete. Moreover, the large heterogeneity of the study groups with multiple CKD etiologies and treatments presents a challenge to a comparative study. However, strong associations between consecutive CKD stages and gradually increasing disturbances of the FA profile suggest that it is CKD itself that is responsible for the observed FA changes. Moreover, our observational analysis is coupled with well-designed cell culture experiments that support our hypothesis. 

Finally, the studied cohort is relatively small, but even with these patient numbers the presented associations among evaluated variables are strong and convincing. 

In conclusion, our study shows that the progressive alterations of serum FA composition in the course of CKD might contribute to CKD related hypertriglyceridemia through influencing the expression of genes of lipid synthesis and release in the hepatocyte.

## 4. Materials and Methods 

### 4.1. Patients

The first part of this study included a cohort of patients with CKD recruited from the Outpatient Unit of the Department of Nephrology, Transplantology and Internal Medicine in the Medical University of Gdansk. The cohort involved 191 patients, aged 18–70, with CKD stages 1–2 (*n* = 40), 3a (*n* = 23), 3b (*n* = 27), 4–5 (*n* = 19), undergoing hemodialysis (*n* = 28), peritoneal dialysis (*n* = 30) and renal transplantation (*n* = 24). The etiology of CKD was diabetes (24 patients), glomerulonephritis (32), hypertension (25), anemia (6), autosomal dominant polycystic kidney disease (13), vasculitis (5), systemic lupus erythematosus (5), obstructive (18) or other (63). Ninety one CKD patients used statins, and four used fibrates. In addition, we recruited a group of control subjects of similar age and sex and free from any kidney damage (*n* = 30). Subjects using FA supplements were excluded from the study. The study was performed in agreement with the principles of the Declaration of Helsinki of the World Medical Association. Experimental protocols received approval from the Local Bioethics Committee at the Medical University of Gdansk (protocol no. NKEBN/614/2013-2014 issued on 28 May 2014) and informed consents were obtained from patients and healthy volunteers. All laboratory tests were carried out at the Central Clinical Laboratory of the Medical University of Gdansk.

The CKD stage assessment was based on the estimated glomerular filtration rate (eGFR) calculated with the CKD-EPI formula [26]. Presence of cardiovascular disease (CVD), diabetes mellitus (DM) and hypertension was based on the medical records. Body mass index (BMI) was calculated as the body mass divided by the square of the body height, and homeostatic model assessment for insulin resistance (HOMA IR) as glucose concentration (mg/dL) multiplied by insulin (µU/mL) and divided by 405. Blood was donated by the patients and the controls after an overnight fast. Dietary habits were investigated with the use of FFQ6 (food frequency questionnaire), the most common dietary assessment tool used in large epidemiologic studies of diet and health and validated for the Polish population [27]. FFQ is a tool for the evaluation of frequency (times/person/day) and amount (g/person/day) of food consumed during a year. The frequency of product consumption is determined by respondents by free pointing to habitual intake frequency from a list of 55 line items where each line item is defined by a series of foods or beverages.

### 4.2. Lipid Extraction and FAME Analysis

Total lipids in serum were extracted using the method described by Folch et al. [28]. Lipid samples were hydrolyzed with 0.5 M KOH in methanol and FA methyl esters (FAMEs) were prepared using 10% Boron Trifluoride reagent (BF3/methanol). After the evaporation of n-hexane, FAMEs were dissolved in dichloromethane and analyzed on a GC-MS QP-2010 SE (SHIMADZU Kioto, Japan) [29]. Compounds were separated on a 30 m × 0.25 mm i.d., HP-5 capillary column (film thickness 0.25 μm). The temperature of the column was programmed from 60 °C to 300 °C at a rate of 4 °C min^−1^ with helium as carrier gas at a column head pressure of 60 kPa. 19-methyl-eicosanoate was used as the internal standard. All the chemicals and reagents were obtained from Sigma-Aldrich (Saint Louis, MO, USA). Exemplar chromatogram of GCMS fatty acid analysis from CKD patient is presented on supplementary Figure S1.

### 4.3. Cell Cultures and Treatment

The second part of the study involved in vitro and ex vivo experiments on the HepG2 human hepatoma cell line (ECACC, Salisbury, UK). Liver is the major organ responsible for lipid metabolism. Hep G2 cells are derived from liver tissue of human and are widely used as a model system for studies of liver metabolism. These cells secrete a variety of liver derived plasma proteins, and express the genes of lipid metabolism [30,31]. Cells were cultured in a humidified incubator at 37 °C with 5% CO_2_. HepG2 cells were maintained in minimum essential medium Eagle (MEM) containing 10% heat-inactivated fetal bovine serum (FBS), 2 mM L-glutamine, 1% non-essential amino acids, 100 units/mL penicillin and 100 mg/mL streptomycin. The medium was replenished every two days. 

Hepatocytes were treated with the following: OA, AA, DHA or FA extracted from a mix of serums obtained from control subjects and FA extracted from a mix of serums obtained from CKD patients with stage 5. OA, AA and DHA were purchased from Sigma-Aldrich (Saint Louis, MO, USA). FA preparations from patients and controls were prepared on site. From 12 randomly selected samples of healthy controls and 12 CKD stage 5 patients, 200 µL of serum was taken and two solutions were prepared (control-mix and CKD-mix). Total lipids were extracted and hydrolyzed with 0.5 M KOH in methanol exactly as the samples for GC-MS analysis of FA but FA were not derivatized. Experimental stock solutions were prepared 48 h prior to experiments. All test components were conjugated with bovine serum albumin (BSA). Briefly, FA was dissolved in ethanol at 70 °C, then mixed with 10% BSA at 55 °C for 10 min to create conjugates. Cells were treated with FA conjugates dissolved in a serum-free medium. On the day after the passage, the cells were washed twice with 1 mL of phosphate-buffered saline (PBS), and then the specified experimental medium was added. Control cells were run in parallel in medium without test components. After 48 h incubation, the cells were washed twice with 1 mL of PBS and used for RNA isolation. OA, AA and DHA were tested in three variants of concentration of 25 uM, 50 uM and 100 uM. Concentration of FA mixes was adjusted to the concentrations observed in a patient’s serum.

Total cellular RNA was isolated from HepG2 by GenElute Mammalian Total RNA Miniprep Kit (Sigma-Aldrich, (Saint Louis, MO, USA). The total RNA yields were determined for each sample by automated gel electrophoresis (Experion, Bio-Rad, Hercules, CA, USA). cDNA was synthesized from 1 μg of total RNA using random hexamers and RevertAid reverse transcriptase according to the manufacturer’s instructions. An aliquot of each cDNA synthesis reaction was subjected to PCR amplification using a CFX Connect Real-Time System (Bio-Rad, Hercules, CA, USA). The primers sequences are presented in supplemental Table S2. Results were normalized against a combination of β-actin and cyclophilin A.

### 4.4. Triacylglycerols Content Analysis

TAG content was determined in the HepG2 culture media using a triglyceride colorimetric assay kit (Cayman Chemical, Ann Arbor, MI, USA). Triacylglycerols in the samples were hydrolyzed by lipase to produce glycerol and free fatty acids. Released glycerol was subsequently measured by a coupled enzymatic reaction system (glycerol kinase, glycerol phosphate oxidase, peroxidase) that produce a brilliant purple quinoneimine dye. All samples and standards were measured in duplicate. The absorbance was measured at 540 nm.

### 4.5. Statistical Analyses

Differences across the patient/control groups or cell experiments were analyzed, as appropriate, by a t-test or analysis of variance (ANOVA) followed by a post-hoc correction (Bonferroni) in the case of multi-group comparisons. To search for independent relationships between FA content and CKD, a general linear model (GLM) was applied. Statistical processing of the results was performed with the use of the statistical software STATISTICA PL v 13.0 (Statsoft, Kraków, Poland).

## Figures and Tables

**Table 1:** Selected biochemical and anthropometric characteristics of the study subjects. * statistically significant comparing to controls at *p* < 0.05; # *p* < 0.01. CKD—chronic kidney disease, HD – hemodialysis, PD—peritoneal dialysis, Tx—renal transplantation, eGFR—glomerular filtration rate, BUN—blood urea nitrogen, TAG—triacyloglycerol, CRP—C-reactive protein, BMI—body mass index, HOMA IR—Homeostatic model assessment for insulin resistance, DM—diabetes mellitus, CVD—cardiovascular disease, HP—hypertension.

Item	Healthy Control	CKD 1-2	CKD 3a	CKD 3b	CKD 4-5	HD	PD	Tx
Age (years)	55.57 ± 1.34	53.65 ± 2.30	60.57 ± 2.32	61.15 ± 2.64	61.00 ± 2.81	60.14 ± 2.40	51.27 ± 2.18	52.17 ± 2.14
Hemoglobin (g/dL)	14.37 ± 0.20	14.23 ± 0.21	14.61 ± 0.30	13.24 ± 0.39 *	12.33 ± 0.39 ^#^	10.71 ± 0.26 ^#^	10.99 ± 0.29 ^#^	13.17 ± 0.41 ^#^
Creatinine (mg/dL)	0.85 ± 0.03	0.83 ± 0.03	1.33 ± 0.04 ^#^	1.72 ± 0.08 ^#^	3.98 ± 0.96 ^#^	7.61 ± 0.46 ^#^	9.55 ± 0.67 ^#^	1.57 ± 0.17 ^#^
eGFR (ml/min/1.73m^2^)	89.07 ± 2.18	88.05 ± 2.49	50.96 ± 1.17 ^#^	36.59 ± 1.06 ^#^	20.63 ± 1.35 ^#^	7.11 ± 0.54 ^#^	6.30 ± 0.71 ^#^	39.27 ± 2.65 ^#^
BUN (mmol/L)	15.92 ± 0.67	16.49 ± 0.64	21.63 ± 0.88 ^#^	31.69 ± 1.94 ^#^	48.34 ± 3.49 ^#^	53.81 ± 3.00 ^#^	57.68 ± 3.54 ^#^	31.32 ± 3.29 ^#^
TAG (mg/dL)	129.80 ± 10.83	148.10 ± 17.07	151.48 ± 14.48	154.77 ± 12.06	161.32 ± 15.32	185.46 ± 32.68	187.41 ± 14.60 ^#^	215.33 ± 37.46 *
Total cholesterol (mmol/L)	211.23 ± 8.19	212.85 ± 7.32	202.22 ± 11.68	199.62 ± 10.33	212.84 ± 12.78	184.25 ± 10.04 *	209.79 ± 12.16	217.46 ± 11.62
HDL cholesterol (mmol/L)	54.30 ± 2.58	55.93 ± 2.20	48.43 ± 2.66	48.73 ± 3.12	48.26 ± 3.53	41.46 ± 2.47 ^#^	38.76 ± 1.92 ^#^	51.71 ± 3.92
LDL cholesterol (mmol/L)	131.10 ± 7.41	126.29 ±6.28	121.32 ± 8.79	119.96 ± 9.30	131.79 ± 10.79	111.67 ± 9.87	135.43 ± 10.90	125.80 ± 10.87
CRP (mg/L)	2.80 ± 0.90	3.38 ± 0.65	3.38 ± 0.77	4.10 ± 0.91	4.59 ± 1.43	14.19 ± 5.43 *	9.05 ± 3.45	8.64 ± 3.98
Albumin (g/L)	39.46 ± 0.45	38.70 ± 1.06	39.52 ± 0.61	37.78 ± 0.76	36.74 ± 1.06 *	31.96 ± 0.68 ^#^	31.60 ± 0.84 ^#^	37.92 ± 0.86
Glucose (mg/dL)	102.55 ± 4.44	102.67 ± 4.79	114.00 ± 5.35	123.07 ± 10.65	125.16 ± 11.34 *	112.96 ± 12.13	96.83 ± 4.95	116.17 ± 10.10
Insulin (µU/mL)	11.77 ± 0.96	12.99 ± 1.17	15.80 ± 3.39	15.84 ± 1.19 *	21.05 ± 6.99	23.55 ± 9.47	15.13 ± 4.20	30.18 ± 15.00
Na^+^ (mmol/L)	140.27 ± 0.42	140.00 ± 0.41	140.10 ± 0.34	139.62 ± 0.62	140.68 ± 0.78	137.25 ± 0.56 ^#^	141.17 ± 0.46	139.54 ± 0.68
K^+^ (mmol/L)	4.36 ± 0.05	4.24 ± 0.05	4.50 ± 0.09	4.52 ± 0.09	4.81 ± 0.09 ^#^	5.09 ± 0.15 ^#^	4.42 ± 0.10	4.26 ± 0.08
BMI (kg/m^2^)	27.52 ± 0.67	27.52 ± 0.84	28.63 ± 1.05	31.16 ± 1.13 *	27.54 ± 1.18	25.84 ± 1.15	26.16 ± 0.77	27.04 ± 0.85
HOMA IR	1.93 ± 0.42	3.13 ± 0.40 *	3.73 ± 1.29	4.32 ± 0.53 ^#^	6.14 ± 3.57	5.49 ± 2.08 *	4.44 ± 1.98	7.15 ± 2.65 *
Concomitant diseases (%)								
Diabetes mellitus (DM)	3.33 ± 3.33	12.50 ± 5.30	27.27 ± 9.50 *	37.04 ± 9.47 ^#^	42.11 ± 11.64 ^#^	35.71 ± 9.22 ^#^	16.67 ± 6.92	33.33 ± 9.83 ^#^
Cardiovascular disease (CVD)	3.33 ± 3.33	15.00 ± 5.72	40.91 ± 10.49 ^#^	44.44 ± 9.75 ^#^	42.11 ± 11.64 ^#^	57.14 ± 9.52 ^#^	36.67 ± 8.95 ^#^	33.33 ± 9.83 ^#^
Hypertension (HP)	33.33 ± 8.75	70.00 ± 7.34 ^#^	95.65 ± 4.45 ^#^	92.59 ± 5.14 ^#^	100.00 ±7.65 ^#^	92.86 ± 4.96 ^#^	100 ± 0.00 ^#^	100.00 ± 0.00 ^#^

**Table 2:** The percent content of the main classes of fatty acids in serum of patients at successive stages of chronic kidney disease (CKD). The data are presented as fatty acid proportions (%). * statistically significant comparing to controls at *p* < 0.05; # *p* < 0.01. SFA—saturated fatty acids, MUFA—monounsaturated fatty acids, PUFA—polyunsaturated fatty acids.

Item	Healthy Control	CKD 1-2	CKD 3a	CKD 3b	CKD 4-5	HD	PD	Tx
14:0	1.18 ± 0.06	1.18 ± 0.06	1.26 ± 0.07	1.18 ± 0.08	1.06 ± 0.06	1.17 ± 0.08	0.98 ± 0.06 *	1.33 ± 0.12
16:0	23.00 ± 0.33	23.04 ±0.30	23.64 ± 0.50	23.21 ± 0.30	23.50 ± 0.39	23.51 ± 0.39	22.79 ± 0.37	23.86 ± 0.45
18:0	7.23 ± 0.12	7.15 ± 0.09	7.12 ± 0.16	7.02 ± 0.16	6.79 ± 0.18 *	7.04 ± 0.20	6.87 ± 0.18	6.72 ± 0.13 ^#^
Other SFA	1.57 ± 0.04	2.08 ± 0.09 ^#^	1.91 ± 0.11 ^#^	1.98 ± 0.11 ^#^	2.00 ± 0.11 ^#^	2.04 ± 0.10 ^#^	1.48 ± 0.07	2.27 ± 0.10 ^#^
Total SFA	32.97 ± 0.34	33.44 ± 0.36	33.93 ± 0.14	33.38 ± 0.44	33.36 ± 0.49	33.75 ± 0.38	32.12 ± 0.51	34.18 ± 0.57
14:1	0.07 ± 0.01	0.07 ± 0.01	0.08 ± 0.01	0.07 ± 0.01	0.06 ± 0.01	0.07 ± 0.01	0.05 ± 0.00 ^#^	0.08 ± 0.01
16:1	1.93 ± 0.30	2.80 ± 0.17 ^#^	2.41 ± 0.32	2.85 ± 0.17 ^#^	2.22 ± 0.35	2.78 ± 0.22 *	2.76 ± 0.15 *	3.06 ± 0.17 ^#^
18:1	26.04 ± 0.55	26.04 ± 0.47	25.68 ± 1.29	27.56 ± 0.59	28.57 ± 0.49 ^#^	29.24 ± 0.75 ^#^	29.81 ± 0.58 ^#^	28.00 ± 0.64 *
Other MUFA	0.47 ± 0.02	0.46 ± 0.01	0.47 ± 0.02	0.51 ± 0.02	0.57 ± 0.07	0.53 ± 0.02	0.47 ± 0.02	0.53 ± 0.03
Total MUFA	29.60 ± 0.64	29.59 ± 0.54	30.39 ± 0.65	31.09 ± 0.62	32.19 ± 0.54 ^#^	32.81 ± 0.87 ^#^	33.10 ± 0.68 ^#^	31.67 ± 0.73 *
18:3n-3	0.35 ± 0.02	0.30 ± 0.02	0.29 ± 0.03	0.27 ± 0.02 ^#^	0.24 ± 0.03 ^#^	0.20 ± 0.02 ^#^	0.24 ± 0.02 ^#^	0.21 ± 0.02 ^#^
20:5n-3	1.11 ± 0.13	0.85 ± 0.07	0.81 ± 0.07	0.77 ± 0.07 *	0.77 ± 0.11	0.72 ± 0.07 *	0.95 ± 0.05	0.60 ± 0.06 ^#^
22:6n-3	1.14 ± 0.08	1.01 ± 0.06	1.03 ± 0.08	1.06 ± 0.06	0.96 ± 0.10	0.92 ± 0.09	1.38 ± 0.08 *	0.88 ± 0.08 *
Other n-3 PUFA	0.39 ± 0.00	0.36 ± 0.01	0.34 ± 0.02 *	0.37 ± 0.02	0.34 ± 0.01 ^#^	0.36 ± 0.01	0.50 ± 0.02 ^#^	0.32 ± 0.02 ^#^
Total n-3 PUFA	2.99 ± 0.21	2.52 ± 0.13	2.47 ± 0.15	2.48 ± 0.13	2.31 ± 0.20 *	2.19 ± 0.16 ^#^	3.08 ± 0.13	2.01 ± 0.13 ^#^
18:2n-6	24.90 ± 0.64	25.14 ± 0.60	24.31 ± 0.99	23.85 ± 0.76	23.99 ± 0.95	22.25 ± 0.64 ^#^	22.73 ± 0.67 *	24.13 ± 0.82
20:4n-6	5.56 ± 0.20	5.05 ± 0.17	4.93 ± 0.22 *	5.11 ± 0.17	4.66 ± 0.27 ^#^	4.93 ± 0.25	5.15 ± 0.20	4.03 ± 0.17 ^#^
Other n-6 PUFA	1.44 ± 0.04	1.37 ± 0.04	1.30 ± 0.04 *	1.23 ± 0.05 ^#^	1.10 ± 0.07 ^#^	1.13 ± 0.06 ^#^	1.43 ± 0.04	1.12 ± 0.04 ^#^
Total n-6 PUFA	31.90 ± 0.67	31.57 ± 0.67	30.54 ± 0.24	30.19 ± 0.77	29.72 ± 0.90	28.31 ± 0.72 ^#^	29.31 ± 0.77 *	29.28 ± 0.91 *

**Table 3:** Content of fatty acids in extracts from patients with chronic kidney disease (CKD) stage 5 and healthy controls serum mixes used for hepatocyte treatment in vitro. The data are presented as a fatty acid proportion (%). SFA—saturated fatty acids, MUFA—monounsaturated fatty acids, PUFA—polyunsaturated fatty acids.

Item	Control	CKD Stage 5
14:0	1.02	1.33
16:0	22.72	26.84
18:0	7.99	6.46
Other SFA	1.56	1.10
Total SFA	32.27	35.73
14:1	0.04	0.09
16:1	1.74	3.29
18:1	23.59	29.59
Other MUFA	0.80	0.62
Total MUFA	26.17	33.85
18:3n-3	0.23	0.20
20:5n-3	0.94	0.66
22:6n-3	1.17	0.93
Other n-3 PUFA	0.37	0.30
Total n-3 PUFA	2.71	2.09
18:2n-6	30.03	22.76
20:4n-6	6.03	4.28
Other n-6 PUFA	1.5	0.93
Total n-6 PUFA	37.56	27.97

## Supplementary Materials

Supplementary materials can be found at https://www.mdpi.com/1422-0067/20/10/2470/s1.

## Abbreviations

AA	Arachidonic acid
ACC	Acetyl-CoA carboxylase
ApoA1	Apolipoprotein A1
ApoB	Apolipoprotein B
BMI	Body mass index
BSA	Bovine serum albumin
BUN	Blood urea nitrogen
CDC	Chronic kidney disease
CRP	C-reactive protein
CVD	Cardiovascular disease
DAG	diacylglycerol
DHA	Docosahexaenoic acid
DGAT	Diacylglycerol O-transferase
DM	Diabetes mellitus
eGFR	Glomerular filtration rate
ELOVL	Elongase
FA	Fatty acids
FAME	Fatty acid methyl ester
FASN	Fatty acid synthase
FFQ	Food frequency questionnaire
GLM	General linear model
HD	Hemodialysis
HNF4α	Hepatocyte nuclear factor 4α
HP	Hypertension
HOMA IR	Homeostatic model assessment for insulin resistance
LXR	Liver-x receptors
MTTP	Microsomal TAG transfer protein
MUFA	Monounsaturated fatty acids
NFκB	Nuclear factor κB
OA	Oleic acid
PD	Peritoneal dialysis
PUFA	Polyunsaturated fatty acids
PPAR	Peroxisome proliferator-activated receptors
SCD	Stearoyl-CoA desaturase
SFA	Saturated fatty acids
SREBP	Sterol regulatory binding proteins
TAG	Triacylglycerol
Tx	Renal transplantation
VLDL	Very low density lipoprotein.
