# Supplementary material for: Alterations of Fatty Acid Profile May Contribute to Dyslipidemia in Chronic Kidney Disease by Influencing Hepatocyte Metabolism
Source: Int J Mol Sci. 2019 May 18;20(10):2470. doi: 10.3390/ijms20102470 (PMC6566623; doi:10.3390/ijms20102470)
Supplement: Supplementary file 1 [file ijms-20-02470-s001.pdf]

Supplementary Materials

Alterations of Fatty Acid Profile May Contribute to Dyslipidemia in Chronic Kidney Disease by Influencing Hepatocyte Metabolism

Aleksandra Czumaj [1], Tomasz Śledziński [1,*], Juan-Jesus Carrero [2], Piotr Stepnowski [3], Malgorzata Sikorska-Wisniewska [4], Michal Chmielewski [4] and Adriana Mika [1,3]

[1] Department of Pharmaceutical Biochemistry, Faculty of Pharmacy, Medical University of Gdansk, 80-211 Gdansk, Poland; aczumaj@gumed.edu.pl (A.C.); adriana.mika@ug.edu.pl (A.M.)
[2] Medical Epidemiology and Biostatistics (MEB), Karolinska Institutet, SE-171 77 Stockholm, Sweden; Juan.Jesus.Carrero@ki.se
[3] Department of Environmental Analysis, Faculty of Chemistry, University of Gdansk, 80-308 Gdansk, Poland; piotr.stepnowski@ug.edu.pl
[4] Department of Nephrology, Transplantology and Internal Medicine, Faculty of Medicine, Medical University of Gdansk, 80-214 Gdansk, Poland; gosia.sikorska@gmail.com (M.S.-W.); chmiel@gumed.edu.pl (M.C.)
* Correspondence: tomasz.sledzinski@gumed.edu.pl; Tel.: +48583491479; Fax: +48583491465

Received: 11 April 2019; Accepted: 17 May 2019; Published: date

Table S1. The patients subjective evaluation of frequency of consumption of selected products rich in mono- and polyunsaturated fatty acids (the scale is 0-6; 6—very often consumed; 1—very rarely consumed; 0— not consumed). Results presented as mean ± SEM. *—statistically significant compared to healthy control at $p < 0.05$

	Healthy control	CKD 1-2	CKD3a	CKD3b	CKD 4-5	HD	PD	Tx	Source of
Salty snacks	1.93 ± 0.17	1.72 ± 0.31	1.52 ± 0.13	1.38 ± 0.12	1.53 ± 0.13	1.36 ± 0.13	1.63 ± 0.17	1.55 ± 0.17	MUFA, n-6 PUFA
Eggs	3.23 ± 0.11	3.10 ± 0.17	3.17 ± 0.12	3.04 ± 0.12	3.21 ± 0.16	2.82 ± 0.21	3.85 ± 0.16	3.14 ± 0.11	n-6 PUFA
Oil	4.00 ± 0.15	3.59 ± 0.15	3.52 ± 0.27	3.35 ± 0.21	3.83 ± 0.20	3.41 ± 0.22	3.52 ± 0.17	3.43 ± 0.18	MUFA, n-6 PUFA
Butter	3.79 ± 0.23	3.92 ± 0.11	4.09 ± 0.30	3.69 ± 0.31	3.68 ± 0.36	3.95 ± 0.36	3.77 ± 0.29	3.81 ± 0.31	MUFA
Margarine	2.90 ± 0.33	2.36 ± 0.17	2.65 ± 0.37	2.04 ± 0.28	1.72 ± 0.32	2.43 ± 0.40	2.73 ± 0.32	2.62 ± 0.39	n-3 PUFA, n-6 PUFA
Lard	1.83 ± 0.14	1.67 ± 0.27	1.65 ± 0.17	1.58 ± 0.17	1.68 ± 0.21	1.91 ± 0.23	1.41 ± 0.13	1.73 ± 0.16	MUFA
Avocado	1.59 ± 0.15	1.46 ± 0.24	1.48 ± 0.18	1.12 ± 0.08	1.50 ± 0.23	1.14 ± 0.09	1.74 ± 0.22	1.45 ± 0.22	MUFA, n-6 PUFA
Olives	2.07 ± 0.20	1.49 ± 0.13	1.52 ± 0.16	1.32 ± 0.11	1.39 ± 0.18	1.27 ± 0.14	1.67 ± 0.21	1.64 ± 0.19	MUFA, n-6 PUFA
Legumes	2.53 ± 0.15	2.24 ± 0.14	2.22 ± 0.16	2.14 ± 0.18	2.37 ± 0.17	2.16 ± 0.22	2.12 ± 0.16	2.23 ± 0.15	n-3 PUFA
Nuts	2.63 ± 0.18	2.56 ± 0.12	2.52 ± 0.23	1.80 ± 0.16	2.32 ± 0.30	1.59 ± 0.17*	2.27 ± 0.21	2.59 ± 0.23	MUFA, n-3 PUFA, n-6

									PUFA
Seeds	2.14 ± 0.16	2.21 ± 0.15	1.91 ± 0.21	1.68 ± 0.23	2.00 ± 0.24	1.55 ± 0.17	2.26 ± 0.27	2.14 ± 0.26	MUFA, n-3 PUFA
Oily fish	2.63 ± 0.14	2.62 ± 0.15	2.50 ± 0.17	2.60 ± 0.14	2.84 ± 0.17	2.23 ± 0.16	2.30 ± 0.13	2.25 ± 0.21	n-3 PUFA

Table S2. Primer Sequences. ACC—acetyl-coenzyme A carboxylase, FASN—fatty acid synthase, SCD1—stearoyl-CoA desaturase, ELOVL6—fatty acid elongase 6, DGAT1—diacylglycerol O-acyltransferase 1, MTTP—microsomal triglyceride transfer protein, ApoB—apolipoprotein B, ApoA1—apolipoprotein A1, SREBP1—sterol regulatory element-binding protein 1.

Primer Name	Forward Sequence (5`–3`)	Reverse Sequence (5`–3`)
ACC	GCCTGACTTTTGATCCGACC	GTTATCCCCAAACCCAGGCA
FASN	CTCGTTGAAGAACGCATCCA	CGCTCGGCATGGCTATCT
SCD1	AAGAGTGTGTTCGTTGCCACTT	GGTAGTTGTGGAAGCCCTC
ELOVL6	CAAAGCACCCGAACTAGGAG	TGGTGATACCAGTGCAGGAA
DGAT1	GCATCACCACACACCAGTTC	TCGCCTGCAGGATTCTTTAT
MTTP	ACAAGCTCACGTACTCCACTG	TCCTCCATAGTAAGGCCACATC
ApoB	CCTGATGGAACAGATTCAAGA	TGGATCATCAGTGATGGCTTT
ApoA1	GCCTTGGGAAAACAGCTAAACC	AGCTTGCTGAAGGTGGAGGTC
SREBP1	CGGAACCATCTTGGCAACA	GCCGGTTGATAGGCAGCTT

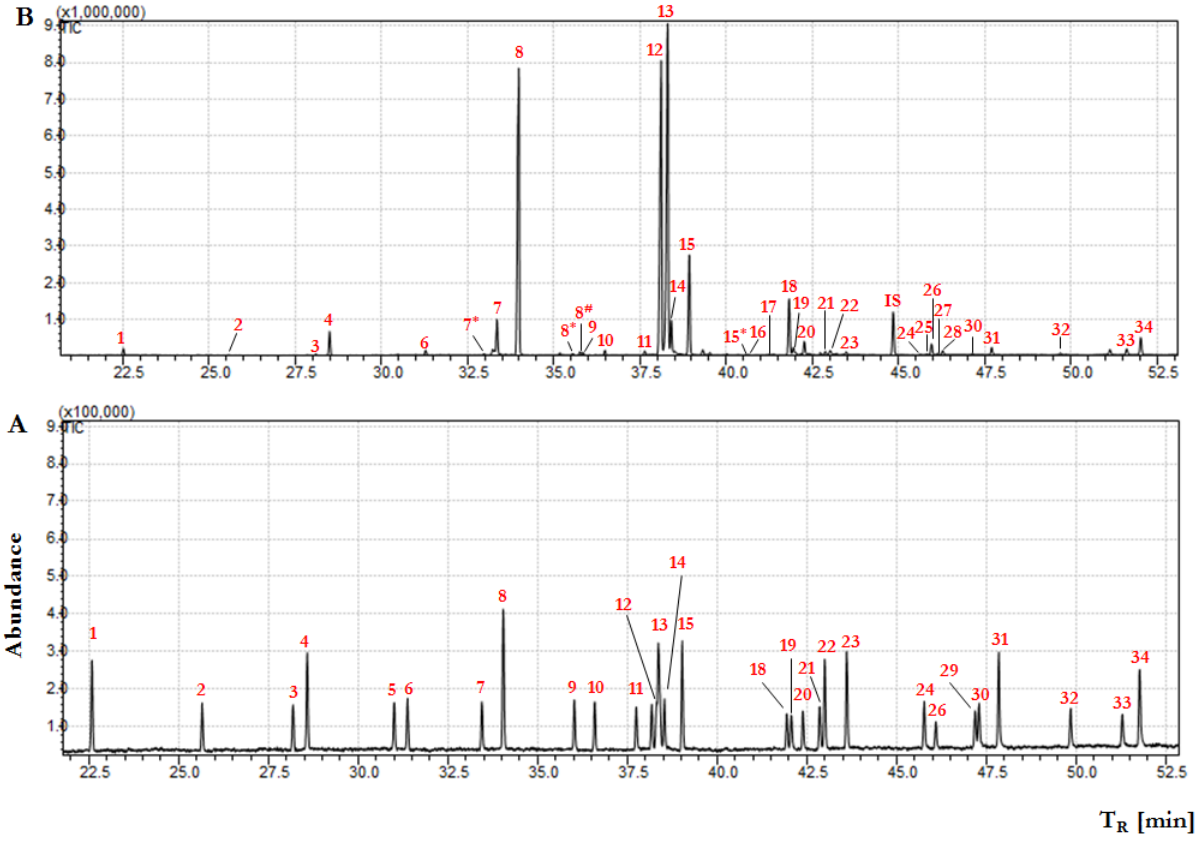

Figure S1. GCMS chromatogram of total ion current of the FAME Supelco® mixture (**A**) and FAME of CKD patient (**B**).

1—C12:0; **2**—C13:0; **3**—C14:1(9c); **4**—C14:0; **5**—C15:1(10c); **6**—C15:0; **7***—iso-C16:0; **7**—C16:1(9c); **8***—iso-C17:0; **8#**—anteiso-C17:0; **8**—C16:0; **9**—C17:1(10c); **10**—C17:0; **11**—C18:3n-3(9c,12c,15c);

12—C18:2n-6(9c,12c); **13**—C18:1(9c); **14**—C18:1(9t); **15**—C18:0; **15***—anteiso-C19:0; **16**—C19:1(10c); **17**—C19:0; **18**—C20:4n-6(5c,8c,11c,14c); **19**—C20:5n-3(5c,8c,11c,14c,17c); **20**—C20:3n-6(8c,11c,14c); **21**—C20:2n-6(11c,14c); **22**—C20:1(11c); **23**—C20:0; **IS**—iso-C21:0; **24**—C21:0; **25**—C22:5n-6(4c,7c,10c,13c,16c); **26**—C22;6n-3(4c,7c,10c,13c,16c,19c); **27**—C22:4n-6(7c,10c,13c,16c); **28**—C22:5n-3(7c,10c,13c,16c,19c); **29**—C22:2n-6(13c,16c); **30**—C22:1(13c); **31**—C22:0; **32**—C23:0; **33**—C24:1(15c); **34**—C24:0.

*—*iso* form of branched chain fatty acids, not present in FAME Supelco® mixture

#—anteiso form of branched chain fatty acids, not present in FAME Supelco® mixture

marked in red in figure legend—fatty acids not present in FAME Supelco® mixture.
